# Big (Sequencing) Future of Non-Coding RNA Research for the Understanding of Cocaine

**DOI:** 10.3389/fgene.2012.00158

**Published:** 2012-09-03

**Authors:** Da-Yu Wu

**Affiliations:** ^1^Division of Basic Neuroscience and Behavior Research, National Institute on Drug Abuse, National Institutes of HealthBethesda, MD, USA

Recent advancement in genomic and genetic sequencing technology has ushered in a new era in which unprecedented amount of genomic sequence and transcript sequence data with extraordinary detail can be generated at incredibly short time. As part of this advancement, the approaches that utilize the powerful next generation sequencing technology, such as RNA-Seq, may have dramatically changed the field of non-coding RNA (ncRNA) research. Since these new approaches do not require prior knowledge of annotated transcripts for probes, theoretically the entire transcriptome of a given sample can be sequenced. This enables the detection of novel transcripts, including both protein coding and ncRNA, as well as RNA with somatic mutations and alternative splicing forms (Trapnell et al., 2009, 2010; Au et al., 2010; Griffith et al., 2010). Before this recent technological advancement, in the more traditional approaches for ncRNA studies, such as using RNA microarray technology, prior sequence knowledge of ncRNA in a cell, tissue, or an organism is required to first generate the microarray. Known RNA and RNA variants of a sample are then hybridized to the array, detected, quantified, and analyzed, while unknown RNA or RNA variants remain undetected and unanalyzed. This is especially limiting for the studying of many classes of ncRNA, such as long ncRNA (lncRNA, which are ncRNA that are longer than 200 nucleotides), due to somatic mutations, epigenetic consequences, and the possibility of secondary structures that longer RNA often take (Wang and Chang, 2011; Mercer et al., 2012; Moran et al., 2012). Some additional advantages of RNA-Seq that have been reported in studying protein coding transcripts, including the capability of detecting high dynamic range of gene expression level, and its high precision and high reproducibility (Marioni et al., 2008; Mane et al., 2009; Bradford et al., 2010; Chen et al., 2010; Twine et al., 2011; Zhang et al., 2012), will likely further ratify this new trend of ncRNA research. Nevertheless, currently microarray is still a valid technology for RNA studies in many other aspects, for its speed, specificities for targeted probing, and its low cost. For example, since statistically 5% of genes give rise to 75% of housekeeping transcripts, much effort is required in RNA-Seq to filter out high abundance transcripts before one can piece together and quantify the interested lower abundance transcripts in a typical RNA sample. Cost is another limiting factor for the migration to RNA-Seq. A typical RNA-Seq run can cost a few thousands, while a typical run for a targeted high throughput microarray costs around hundreds in dollar amount. In the foreseeable future, microarray and RNA-Seq may co-exist and each provides its own aspects of advantage.

If the size of human genome truly reflects the evolution pressure and practical genomic requirements for biological function and survival, one may conclude that the number of genes we have studied and all the facts we have discovered on genes and gene regulation is just the tip of an iceberg, because merely 1.5–2% of nucleotide (nt) bases in the human genome are transcribed into genes that code for proteins (Wolfsberg et al., 2001; The ENCODE Project Consortium, 2007), which has been the main research emphasis for genetic research for the last several decades. With the advancement of modern technologies, including RNA-Seq and microarray technology, it is clear that new information on genomes, transcripts, and their regulation will increase exponentially in the coming decades (Schulz et al., 2008; Zerbino et al., 2012). How to manage, mine, and comprehend these large data will be a real challenge. Meantime the reward can be high and the effort may be well justifiable, considering the likely impact the new knowledge will have on disease research and treatment.

A prominent member of ncRNA in brain function and psychiatric disorder is microRNA (miRNA), which is believed to be the transcripts of 1–3% of the human genome. miRNA is highly involved in brain development and plasticity at the neuronal level (Kapsimali et al., 2007; Choi et al., 2008; Cheng et al., 2009; Liu et al., 2010; Shi et al., 2010; also see reviews by Saba and Schratt, 2010; Konopka et al., 2011; Im and Kenny, 2012). This ncRNA regulates gene expression through RNA interference, RNA degradation, DNA methylation, and chromatin remodeling. As reviewed by two articles in this special issue from Kenny and Mains groups, recent discoveries of miRNA function and mechanisms in cocaine addiction have demonstrated important roles of ncRNA in this psychiatric disorder. For example, in the dorsal striatum, miR-212 was upregulated in rats addicted to cocaine and this upregulation enhances miR-212 regulated CREB signaling and counters the motivational property of cocaine (Hollander et al., 2010). Further studies found this specific function of miR-212 involves its negative homeostatic interaction with transition of acquizition period of addiction to compulsive-like increase of craving of cocaine. The downstream signaling pathway is further revealed in the same study to be through the controlling of BDNF levels in dorsal striatum (Im et al., 2010). Corroborative evidence of cocaine induced various miRNA expression and their functional role in cocaine addiction have also emerged in more and more other laboratories, complementing many aspects of our understanding (Nudelman et al., 2010; Schaefer et al., 2010; Chandrasekar and Dreyer, 2011; Eipper-Mains et al., 2011). Particularly remarkable in one of these studies is that, using RNA-Seq approaches, cocaine is found to both upregulate and downregulate specific miRNA or family of miRNA (Eipper-Mains et al., 2011). While similar findings have been reported earlier in the nervous system (Vo et al., 2005; Krol et al., 2010; Nudelman et al., 2010), this RNA-Seq study is the first comprehensive analysis of cocaine induced changes of miRNA, and has demonstrated the advantage of next generation sequencing technology over microarray technology, which is often weakened by cross hybridization and high background signal, and critically limited by its prerequisite of prior knowledge of ncRNA identity it analyzes.

A much less tapped area in ncRNA research for the understanding of addiction of cocaine, or other substances of abuse, is the lncRNA; Wapinski and Chang, 2011; Huang et al., 2012; Jeggari et al., 2012; Moran et al., 2012. lncRNA are those ncRNA that are longer than 200 nucleotides and are oftentimes transcripts of intergenic regions between transcription clusters, or “foci,” in the genome. Some earlier projects identified around 35,000 long non-coding transcripts from estimated 10,000 distinct loci in mammalian genome, often bearing signatures of protein coding mRNA, such as 5’ capping, splicing, and poly adenylation, but have no apparent open reading frame (ORF) for protein translation (Carninci, 2005, 2006; Kapranov et al., 2010). Remarkably, many more lncRNA may exist but not recognized due to the fact that the majority of lncRNA transcripts may not be poly adenylated (Cheng et al., 2005; Carninci, 2006; Kapranov et al., 2010), while many of the transcripts detection approaches rely on first hybridizing the poly adenylated region. Functionally, lncRNA have been found to have roles in epigenetic regulation, imprinting, and X-chromosome inactivation, in addition to regulation of gene transcription and translation (Mercer et al., 2009; Wang and Chang, 2011; Wapinski and Chang, 2011; Harries, 2012; Huang et al., 2012; Moran et al., 2012). Consequently, lncRNA may play significant roles in cocaine addiction. Indeed, it is likely that future work will identified high numbers of lncRNA in brain areas of the putative learning-reward-addiction neural circuits such as nucleus accumbens, and will likely shed light on important involvement of lncRNA in these brain regions for reward and addiction. How genetic and epigenetic factors interact with these lncRNA, and how changes were brought upon these lncRNA by brain activities, in terms of lncRNA mutation, expression level, dynamics, and function, may provide significant insights on the mechanisms of cocaine addiction. Because substance abuse and dependence involves substantial gene-environment interactions and epigenetic factors, the capability of RNA-Seq in detecting novel gene variations and *de novo* gene mutations also provides valuable and necessary tools for the addiction research field.

The complexity and abundance of ncRNA, especially lncRNA, mandate advanced non-traditional, large scale and high throughput transcript sequencing, and analysis. While microarray technology enjoys established base and proven utility, the next generation sequencing technology represented by RNA-Seq emerges as the future choice approach for the task because of its unbiased coverage of the entire transcriptome of the genome. In addition, RNA-Seq allows versatile experimental design for sequencing depth and adjustable detection sensitivity, using machines of different throughputs and multiplexing different numbers of samples on a sequencing lane. Furthermore, the inherited benefit of RNA-Seq (Mane et al., 2009; Bradford et al., 2010; Chen et al., 2011; Eipper-Mains et al., 2011; also see reviews in this issue), including low background noise, low error rate and low technical variance, the high accuracy of the digital nature, and the capability of detecting novel transcripts and alternative splicing forms, will definitely benefit not only ncRNA analysis, but all other forms of RNA research as well. All these will greatly enable researchers in the field of substance abuse and dependence for future discoveries and breakthroughs.
